# A Rare Case of a Benign Primary Pleomorphic Adenoma of the Lung

**DOI:** 10.7759/cureus.1069

**Published:** 2017-03-02

**Authors:** Venkatkiran Kanchustambham, Swetha Saladi, Setu Patolia, Sara Mahmoud Assaf, David Stoeckel

**Affiliations:** 1 Pulmonary and Critical Care Medicine, Saint Louis University School of Medicine; 2 Internal Medicine, Saint Louis University School of Medicine

**Keywords:** pleomorphic adenoma, lung tumors, salivary gland tumour

## Abstract

Salivary gland tumors (SGT) constitute a small proportion of primary respiratory system neoplasms. Benign SGT comprises pleomorphic adenoma and is exceedingly rare in the lungs. We hereby present a rare case of a benign primary pleomorphic adenoma of the lung. The histological pattern of the tumor was indistinguishable from the head and neck SGT counterparts and showed biphasic morphology. Malignant features were not observed and a metastatic SGT was ruled out. Although little is known about the clinical progression of these rare tumors, surgical resection and interval surveillance remains the treatment of choice.

## Introduction

Pleomorphic adenomas (PA) are the most common type of salivary gland tumors. Histologically, they are characterized by mixed epithelial, myoepithelial and stromal cell components. PA can occasionally undergo a malignant transformation to a carcinoma ex-pleomorphic adenoma and give rise to metastasis. Rarely, they can arise in the tracheobronchial system as primary neoplasm [1]. Pulmonary salivary gland tumors have been the subject of isolated case reports. Herein, we describe a case of an incidental finding of a lung PA in a patient initially admitted for diabetic ketoacidosis. Informed consent was obtained from the patient for this study.

## Case presentation

**Figure 1:**
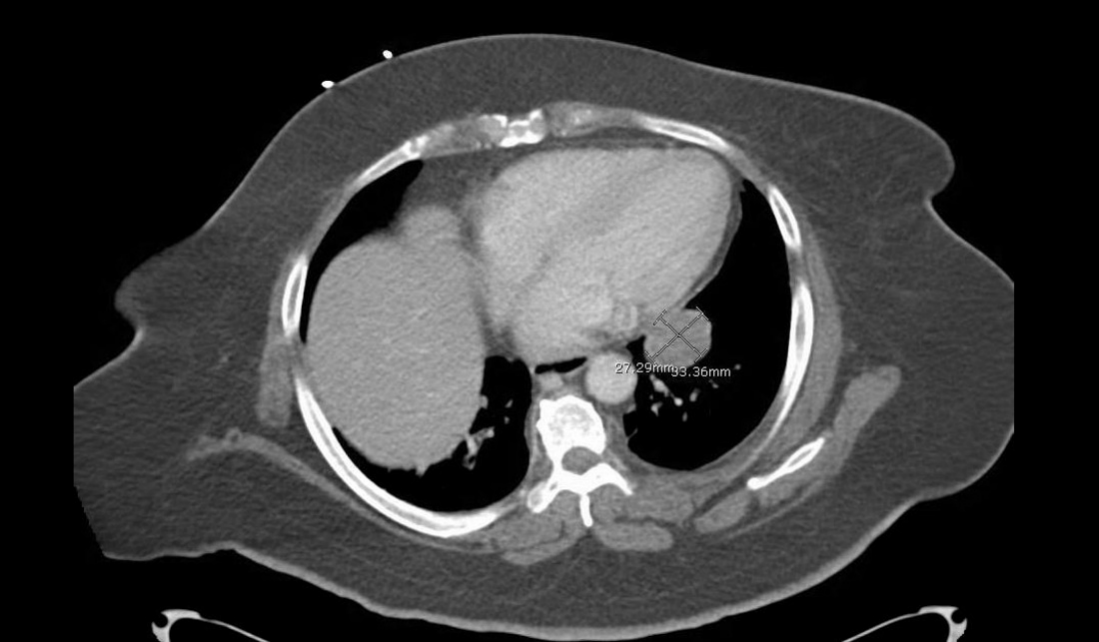
The CT scan of the chest showing a soft tissue attenuating mass in the medial left lower lobe measuring 3.5 x 4.1 x 3.1cm

**Figure 2:**
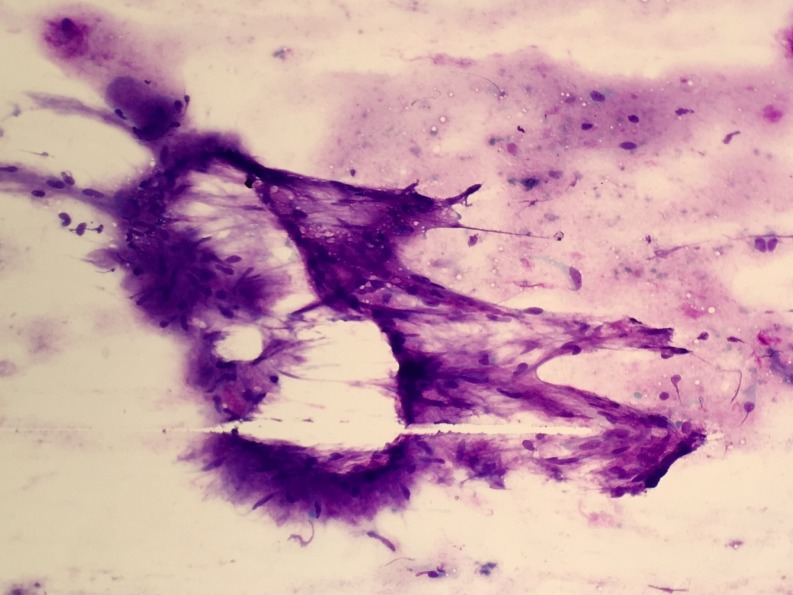
Papanicolaou stained slides showing lymphocytes, benign bronchial and ductal epithelial cells, a myxoid mesenchymal matrix with stromal cells and cartilage

**Figure 3:**
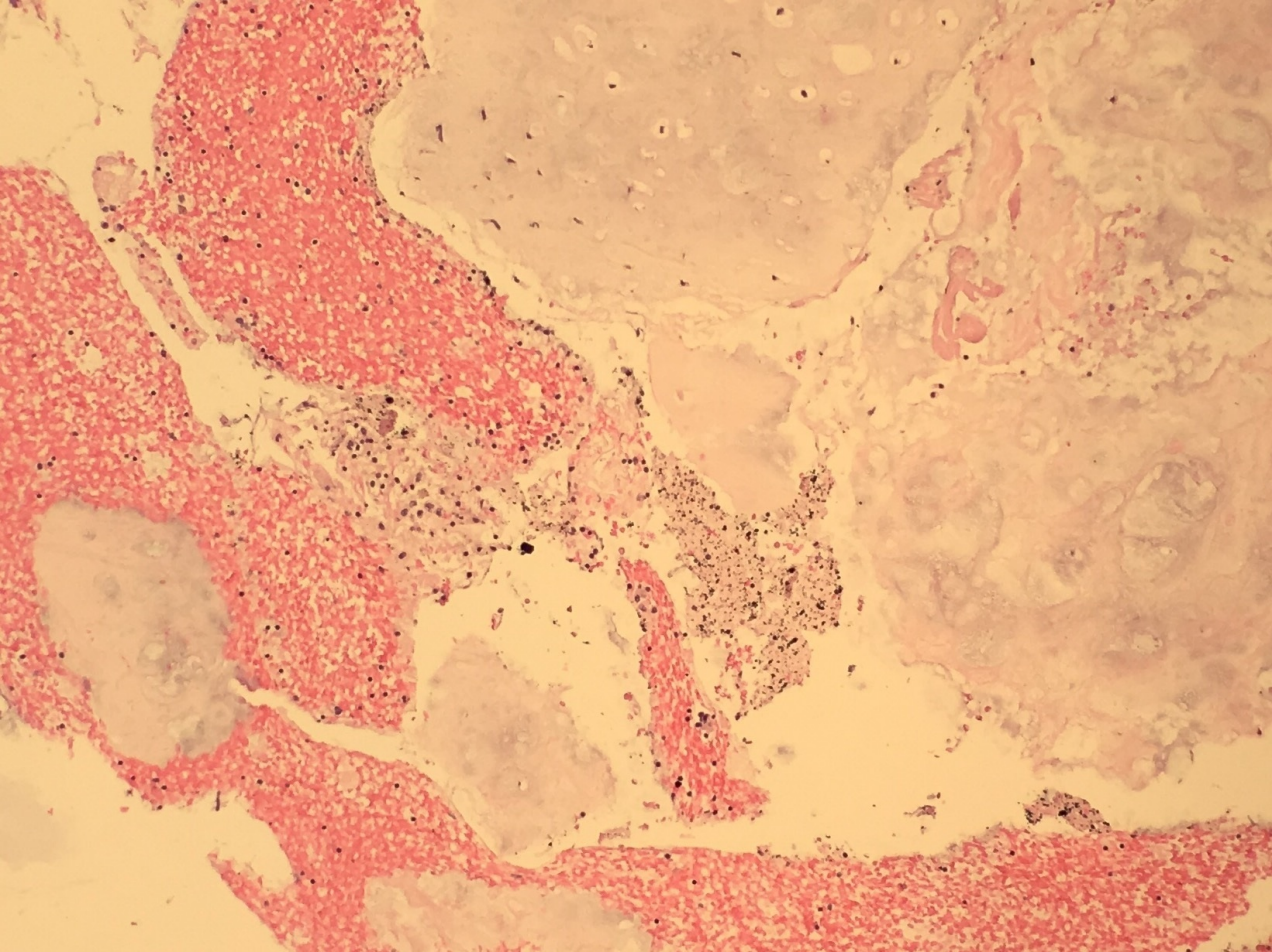
Cell block showing blood, cartilage and rare groups of small uniform epithelial cells

A 63-year-old African American female was admitted to our hospital with nausea, vomiting and abdominal pain of few days duration. She was diagnosed with diabetic ketoacidosis and was started on an insulin drip. Her continuous abdominal pain warranted a computed tomography (CT) of the abdomen and pelvis which revealed an incidental finding of a 3.3 x 2.7cm left lower lobe pulmonary mass, potentially representing a malignant neoplasm. In retrospect, our patient denied any a cough, shortness of breath, hemoptysis or weight loss. A subsequent chest CT scan showed a soft tissue attenuating mass in the medial left lower lobe measuring 3.5 x 4.1 x 3.1cm (Figure 1). A diagnostic bronchoscopy revealed a big left lower lobe mass. Endobronchial ultrasound (EBUS) guided fine needle aspiration (FNA) biopsy of the mass was obtained. A histological examination demonstrated benign bronchial and ductal epithelial cells intermingled with a myxoid mesenchymal matrix, stromal cells, and cartilage (Figures 2-3). No malignant features were identified and diagnosis of benign PA of the lung was subsequently made. After that, a physical examination of the salivary gland was conducted to exclude a metastatic nature of the lung neoplasm and it was normal. The patient was discharged from the hospital with instructions to follow-up with the pulmonary clinic in two weeks.

## Discussion

Pulmonary salivary gland tumors are extremely rare and account for < one percent of all lung neoplasms. A review of the literature, summarized in table 1, showed isolated cases of benign primary lung PA [2-9].

**Table 1:** The case reports of benign primary pleomorphic adenomas of the lungs

Age/Sex	Location	Size	Clinical presentation	Management	Reference
47/female	Left mainstem bronchus	2.5 cm	Cough, chest pain	Pneumonectomy	Moran, et al. 2006
45/female	Left lower lobe	2.5 cm	Incidental finding	Lobectomy	Moran, et al.2006
42/female	Right lower lobe	2.5 cm	Incidental	Lobectomy	Moran, et al. 2006
57/male	Right upper lobe	2 cm	Incidental	Lobectomy	Moran, et al. 2006
58/female	Left upper lobe	2 cm	Productive cough	Lobectomy	Moran, et al.2006
69/female	Left lower lobe	2 cm	Incidental	Lobectomy	Moran, et al. 2006
22/female	Right lower lobe	2 cm	Incidental	Lobectomy	Carretta, et al. 2004
56/female	Right middle lobe	2 cm	Incidental	Lobectomy	Ang KL, et al. 2003
25/female	Left lung periphery	2.5 cm	Incidental	Wedge resection/ VATS	Jin HY, et al. 2007
38/male	Right lobe	10 cm	Post-traumatic after bull injury to the chest	Surgical excision	Pozgain, et al. 2016
67/female	Right middle lobe	NA	Incidental	Lobectomy	Noda M, et al. 2002
36/male	Left lower lobe	NA	Incidental	Lobectomy	Wang JS, et al. 1994
18/female	Right middle lobe	NA	Incidental	Lobectomy	Tanigaki T, et al. 2002

The current world health organization (WHO) classification of lung tumors includes three malignant salivary gland-type tumors: adenoid cystic carcinoma, mucoepidermoid carcinoma, and epithelial-myoepithelial carcinoma. The two benign salivary gland-type tumors are the mucous gland adenomas and pleomorphic adenomas [10]. The latter can arise in the center or periphery of the lung and can grow in size up to 10cm as reported by Pozgain, et al [5].

Macroscopically, PAs are polypoid tumours; they have a rubbery consistency, are often encapsulated and can have a myxoid or cartilaginous appearance. Histologically, they are described as mixed tumors and are characterized by biphasic cellular glandular components, mainly epithelial ducts and/or tubules which are embedded in a chondromyxoid stroma [10].

Most PAs are benign and rarely undergo malignant transformation. Features of malignant variants include cellular atypia, frequent mitotic figures, and necrosis. Our patient’s PA did not have any malignant features. A physical examination of the salivary glands was conducted in our patient to rule out a metastatic origin of the lung PA.

The clinical presentation of PAs varies depending on the location of the tumour. Endobronchial tumours which arise from the epithelium of sub-mucosal bronchial glands are usually associated with cough, hemoptysis and shortness of breath. Peripheral tumours can sometimes present with fever, weight loss and pleural effusions. Nonetheless, the majority of cases are asymptomatic [7]. In fact, our patient denied any respiratory symptoms.

Little is known about the best treatment options for these exceedingly rare lung neoplasms; however surgical resection remains the most common approach as these tumours have the potential of malignant transformation. Our patient is scheduled for a follow-up visit to discuss surgical resection and interval surveillance.

## Conclusions

 In conclusion, primary benign pleomorphic adenomas of the lung are rare tumors of which the majority are asymptomatic and are incidentally discovered. In evaluating patients with pulmonary PAs, metastatic lesions from mixed tumors of the salivary glands and other biphasic pulmonary neoplasms such as blastoma, hamartochondroma, and carcinosarcoma should be considered in the differential diagnosis. Surgical resection is the treatment of choice for PA. Long-term surveillance is warranted due to the concerns for malignant transformation and recurrence of the disease.
